# Protective effect of maternal uteroplacental insufficiency on oxygen-induced retinopathy in offspring: removing bias of premature birth

**DOI:** 10.1038/srep42301

**Published:** 2017-02-14

**Authors:** Silke Becker, Haibo Wang, Baifeng Yu, Randy Brown, Xiaokun Han, Robert H. Lane, M. Elizabeth Hartnett

**Affiliations:** 1 John A. Moran Eye Center, University of Utah, Salt Lake City, UT, USA; 2 Department of Pediatrics, University of Utah, Salt Lake City, Utah, USA; 3 Children’s Hospital of Wisconsin, Milwaukee, Wisconsin, USA

## Abstract

To address the hypothesis that maternal uteroplacental insufficiency (UPI) increases severity of retinopathy of prematurity, we developed a composite rat model of UPI and oxygen-fluctuations and removed premature birth as a confounding factor. Timed-pregnant Sprague-Dawley dams underwent bilateral uterine artery ligation or anesthesia (control) at e19.5. Full-term pups developed in room air (RA) or an oxygen-induced retinopathy (OIR) model. Isolectin-stained retinal flat-mounts were analyzed for percent of areas of avascular/total retina (AVA) and of intravitreal neovascular/total retina (IVNV). Pup weights and serum and mRNA of liver and kidney VEGF, IGF-1, and erythropoietin (EPO) were determined. Multivariable mixed effects linear regressions and Pearson correlations were performed using STATA14. Postnatal growth restriction occurred in pups in UPI/RA, but not in UPI/OIR. Weight gain was similar between UPI/OIR and control/OIR pups. AVA was reduced and a trend toward reduced IVNV was seen in UPI/OIR compared to control/OIR. No difference in birth weights of UPI/OIR vs. control/OIR pups occurred. Serum and renal IGF-1 and EPO were significantly increased in UPI/OIR compared to control/OIR pups. In the absence of prematurity, UPI increased angiogenic factors in association with reduced OIR severity, suggesting that ischemia from UPI could yield protective angiogenic effects by offspring.

Retinopathy of prematurity (ROP) is a leading cause of childhood blindness and is increasing in incidence throughout the world^1^. ROP is closely linked to the degree of prematurity with severe ROP being more common in infants of extremely young gestational ages or low birth weights^2^. The causes of premature birth remain incompletely understood, but one association is with maternal preeclampsia. A component of preeclampsia is uteroplacental insufficiency (UPI) and newborn outcomes, such as postnatal growth, are influenced by UPI^3^. Poor postnatal growth has been linked to severe ROP^4^. However, the clinical literature regarding the risk of ROP from preeclampsia is mixed, with some studies reporting an association between ROP and preeclampsia^5^,^6^ and others finding an apparent “protective” association between the two^7^,^8^. By necessity, clinical studies are observational in design and are difficult to interpret, because of the inherent bias created by the association of preterm birth with both preeclampsia and ROP and the fact that clinical observational studies rarely delineate the specific physiological factors that determine risk for ROP.

Since maternal preeclampsia and particularly UPI can lead to poor infant growth^3^, a group of investigators addressed the hypothesis that maternal preeclampsia increases ROP by removing premature birth as a confounder and focusing on growth restriction in pups. Protein-restricted pregnant dams compared to pregnant dams on a normal diet delivered full-term growth-restricted rat pups. The pups were placed into a controlled variable oxygen environment to create retinopathy similar to some features seen in ROP^9^. The investigators reported increased peripheral avascular retinal areas in growth-restricted pups compared to pups that had normal nourishment^10^. The study suggested that growth restriction, such as what can occur from maternal preeclampsia, initially delays physiologic retinal vascularization and may, thereby, increase the risk of subsequent vasoproliferation into the vitreous, as seen in severe ROP^11^.

In an effort to further understand events surrounding preeclampsia and its risk on ROP, we studied the effect of maternal late-gestation UPI. Full-term pups, born to dams with UPI, were placed into the setting of oxygen fluctuations having similarities to conditions of preterm infants at risk for ROP. We tested the hypothesis that maternal UPI leads to more severe retinal vascular features of ROP in full-term offspring. We found, however, that the combination of maternal UPI and pup oxygen fluctuations led to reduced severity of retinopathy in association with increased circulating growth factors compared to control situations from regulation of factors in the offspring.

## Results

### Characterization and optimization of UPI/OIR model

**Figure 1:**
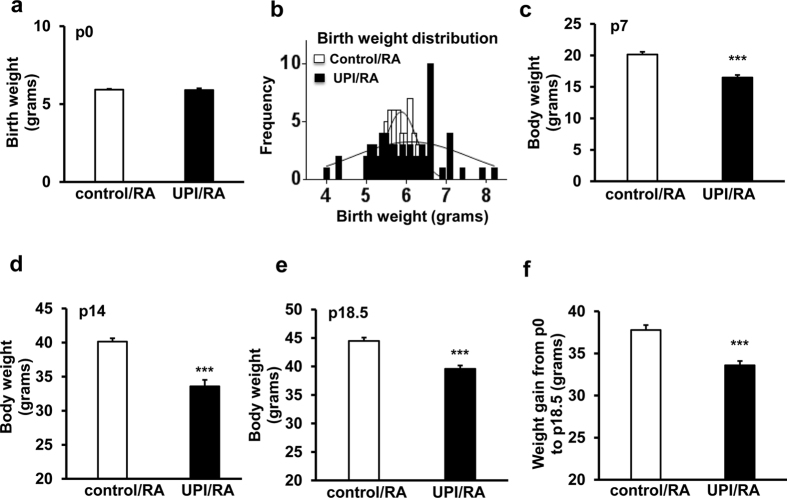
UPI pups have lower weights in room air (RA) at several developmental ages. (**a**) Average birth weight, (**b**) birth weight distribution at postnatal day 0 (p0), (**c**) average body weight at p7, (**d**) average body weight at p14, (**e**) average body weight at p18.5 and (**f**) weight gain from p0 to p18.5 (***p < 0.001 vs. control/RA; n = 11–36).

Bilateral uterine artery ligation in rat dams causes severe UPI and leads to full-term birth of pups two days later^12^. Since sham surgery alone without uterine artery ligation impairs postnatal pup growth, pups born to dams that underwent anesthesia only were used as controls (Erin Zinkhan, personal communication, 2016). We assessed birth weights of pups in UPI and control litters raised in room air (RA). There was no difference in average birth weights between UPI/RA and control/RA litters (Fig. 1a); however, in UPI/RA litters, there was a broader range of pup birth weights compared to control/RA, ranging from 4 g to 8.2 g in UPI/RA pups compared to 5.3 g to 6.9 g in control/RA pups (Fig. 1b). Induced UPI in dams was sufficient to cause growth restriction in pups raised in RA when analyzed at postnatal day (p)7 (Fig. 1c), p14 (Fig. 1d), and p18.5 (Fig. 1e) compared to respective days in controls. Weight gain was also reduced between birth and p18.5 in room-air raised pups born to dams that underwent UPI surgery compared to controls (Fig. 1f). Litter sizes tended to be smaller in dams with UPI (9.25 ± 0.48 pups/litter in 4 litters) compared to control dams (11.75 ± 1.11 pups/litter in 4 litters), but this was not significantly different. Also, nursing and maternal care to pups were similar by dams that had undergone UPI surgery compared to control, and it was previously reported that growth restriction in pups was not differentially affected by milk from UPI dams compared to control dams^13^.

**Figure 2:**
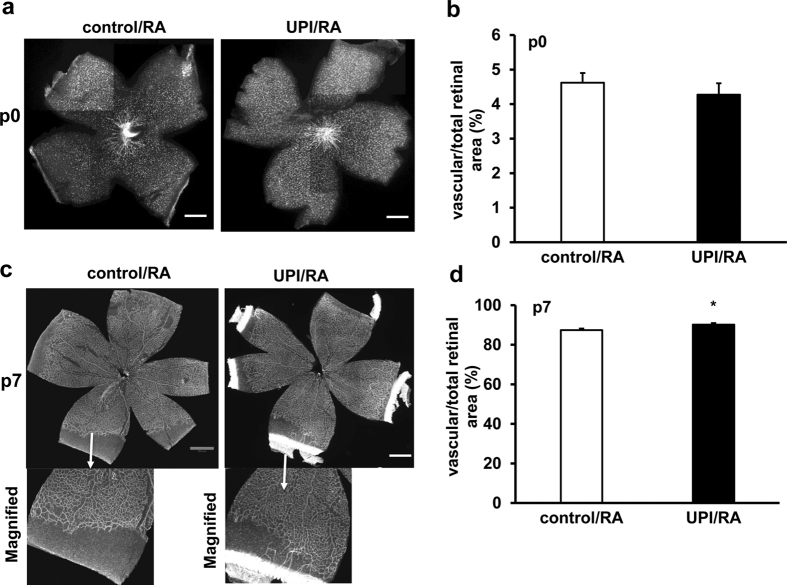
UPI pups show increased physiological retinal vascularization in room air (RA). (**a**) Representative retinal flatmount images (Scale bar, 990 μm) and (**b**) quantification of percent of vascularized retinal area/total retinal area at postnatal day 0 (p0); (**c**) Representative retinal flatmount images (Upper row-Mag, 4X and lower row-Magnified images; Scale bar, 990 μm) and (**d**) quantification of percent of vascularized retinal area/total retinal area at p7 (*p < 0.05 vs. control/RA; n = 9–32).

Retinal vascularization at birth (p0) was similar between control/RA and UPI/RA pups (Fig. 2a,b). The susceptibility of pups to pathologic angiogenesis in OIR is affected by the size (and hypoxic drive) of avascular retina^14^. Therefore, we determined if there were differences in the areas of physiologic retinal vascularization between UPI/RA and control/RA pups at p7, a time point when the retina is still incompletely vascularized in room-air raised rats^15^,^16^. Pups born to dams with UPI had greater vascular/total area than control pups (Fig. 2c,d) and the difference appeared to be related to greater vascularization in female pups (Supplemental Fig. 1). There was no difference in total retinal areas between male and female pups at p7. There was also no difference in vascularized/total retina between male and female pups at p14, when the inner retinal plexus was fully extended.

**Figure 3:**
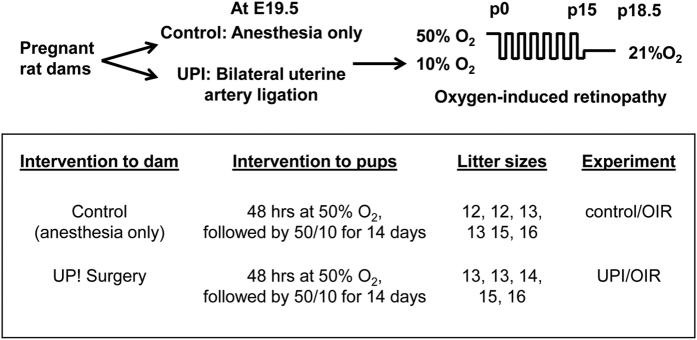
Experimental procedure of composite rat models of uteroplacental insufficiency (UPI) and modified oxygen-induced retinopathy (OIR).

The standard 50/10 oxygen-induced retinopathy model is highly representative of human ROP^9^. In it, newborn rat pups are exposed to fluctuations in inspired oxygen levels between 50% or 10% oxygen every 24 hours for 7 cycles. In order to reduce mortality of pups born to UPI dams and exposed to oxygen fluctuations, the oxygen protocol was modified to include newborn pups in litter sizes of 12–16 pups exposed to 48 hours of 50% O_2_ beginning within 6 hours of birth, followed by 6.5 cycles of oxygen between 10% and 50% O_2_ until day 15. Pup mortality was reduced from 71.4% to 13.6% in the UPI/OIR group (compared to 3.5% in the control/OIR group). Subsequent experiments were performed on litters with 12–16 pups using this modified oxygen-induced retinopathy model, hereafter called OIR (Fig. 3 and Supplemental Table 1). (In pups born to dams without UPI, this modified model, i.e., OIR, did not have different percents of avascular/total retinal area or of intravitreal neovascular/total retinal areas than the standard 50/10 oxygen-induced retinopathy model, Supplemental Fig. 2).

### OIR reduces Pup Growth in UPI and control

**Figure 4:**
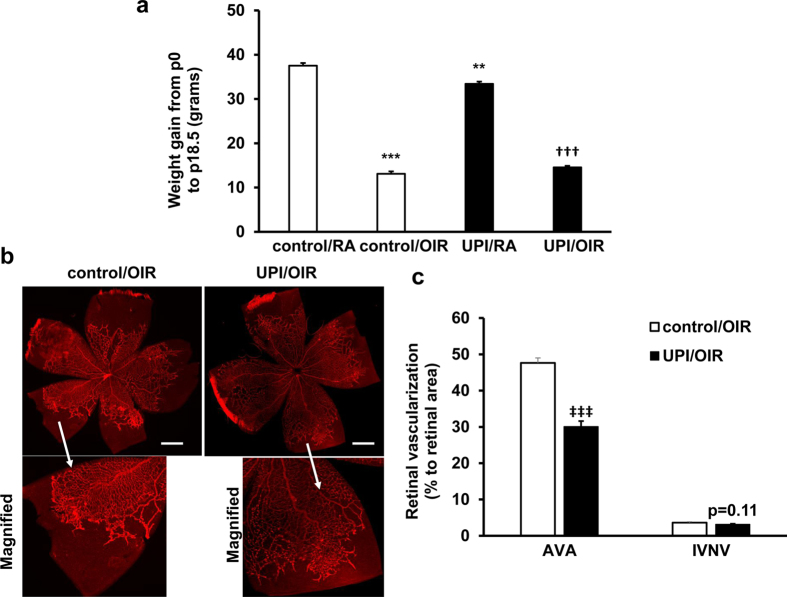
Both control/OIR pups and UPI/OIR pups have reduced postnatal growth and UPI/OIR pups have reduced AVA compared to control/OIR pups. (**a**) Weight gain from postnatal day 0 (p0) to p18.5 was measured in control and UPI pups raised in room air (control/RA and UPI/RA) and OIR model (control/OIR and UPI/OIR) (**p < 0.01, ***p < 0.001 vs. control/RA; ^†††^p < 0.001 vs. UPI/RA; n = 11–56); (**b**) Representative retinal flatmount images (Upper row-Mag, 4X and lower row-Magnified images; Scale bar, 990 μm) and (**c**) quantification of AVA and IVNV at postnatal day 18.5 (p = 0.11 vs. IVNV of control/OIR, ^‡‡‡^p < 0.001 vs. AVA of control/OIR; n = 52–56).

OIR reduced mean weight gain at p18.5 in pups in the control/OIR and UPI/OIR groups compared to respective room air raised pups; however, weight gain was similar between pups in the UPI/OIR and control/OIR groups (Fig. 4a).

### UPI/OIR has less avascular retina and vasoproliferation than control/OIR

We predicted that UPI would cause more severe features of OIR in pups, namely larger areas of percent avascular/total retinal area (AVA) and of percent intravitreal neovascular/total retinal area (IVNV) based on the literature that severe ROP occurs more frequently in growth restricted infants^17^ and a previous study in which experimentally induced growth restriction from protein malnutrition increased AVA^10^. Using multivariable mixed effects linear regressions to account for clustering effects within litters, we unexpectedly found that pups from UPI/OIR had significantly reduced AVA at p18.5 compared to control/OIR (Fig. 4b,c). To determine if anesthesia alone affected AVA, we performed a separate experiment comparing control/OIR to pups born to dams with no anesthesia and placed into the OIR model. There was no difference in AVA between the control/OIR and the no anesthesia/OIR groups (Supplemental Fig. 3). Aligned with the finding of reduced AVA in UPI/OIR retinas, there was also a trend toward reduced IVNV in UPI/OIR compared to control/OIR (Fig. 4b,c, p = 0.11). Reduced AVA and a pattern toward reduced IVNV in UPI/OIR were similar for both male and female pups.

### Birth weight is not associated with retinal vascular features UPI/OIR pups

**Figure 5:**
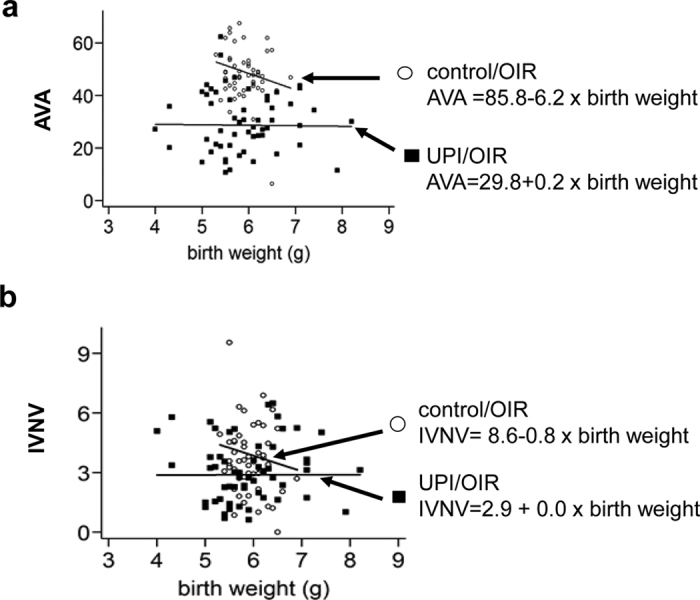
Correlation of birth weight to AVA and IVNV. Correlation between birth weight (**a**) with AVA, and (**b**) with IVNV. Slopes vary but no significance was found.

To assess if reduced AVA in UPI/OIR was related to birth weight, we correlated birth weight and AVA or IVNV in control/OIR pups and UPI/OIR pups. There was a pattern for AVA and IVNV to be greater in control/OIR pups with low birth weights (Fig. 5a), in line with clinical reports of severe ROP in infants associated with low birth weight^4^. However, the same relationship between birth weight and retinal vascular features of OIR was not found in UPI/OIR pups (Fig. 5b), and no correlation was found significant.

### Association of Growth and Angiogenic Factors in UPI/OIR

**Figure 6:**
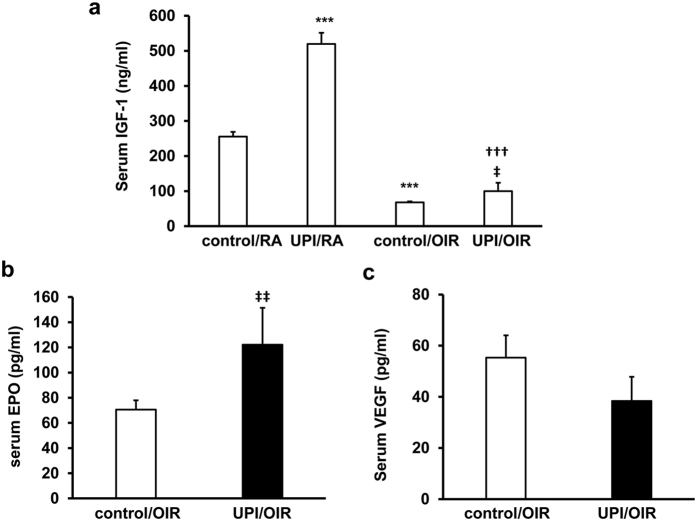
UPI pups have greater circulating growth factors. (**a**) ELISA of IGF-1 in serum from control/RA, UPI/RA, control/OIR and UPI/OIR pups (***p < 0.001 vs. control/RA; ^†††^p < 0.001 vs. UPI/RA; ^‡^p < 0.05 vs. control/OIR); (**b**) ELISA of EPO and (**c**) ELISA of VEGF in serum from control/OIR and UPI/OIR pups at postnatal day 18.5 (p18.5) (^‡‡^p < 0.01 vs. control/OIR; n = 4–28).

Low serum IGF-1 has been associated with reduced infant growth, increased avascular retina and subsequent severe ROP in human premature infants^18^. Intrauterine growth restriction in pups induced by bilateral uterine artery ligation in dams is associated with epigenetic regulation of hepatic IGF-1 expression^19^ and altered expression of hippocampal estrogen and estrogen receptor alpha^20^. We, therefore, were interested in whether UPI/OIR affected IGF-1 levels in pups. We measured serum IGF-1 in UPI/OIR, control/OIR, UPI/RA and control/RA pups. Compared to control/OIR, pups in the UPI/OIR group had higher circulating IGF-1 levels in the serum (Fig. 6a). These UPI/OIR pups also appeared to experience catch-up growth compared to control/OIR (Fig. 4a). However, both UPI/OIR and control/OIR pups had lower serum IGF-1 compared to respective groups of RA pups. In UPI/RA, serum IGF-1 was significantly greater than in the control/RA pups (Fig. 6a), even though UPI/RA pups had poorer postnatal growth compared to control/RA (Fig. 1).

**Figure 7:**
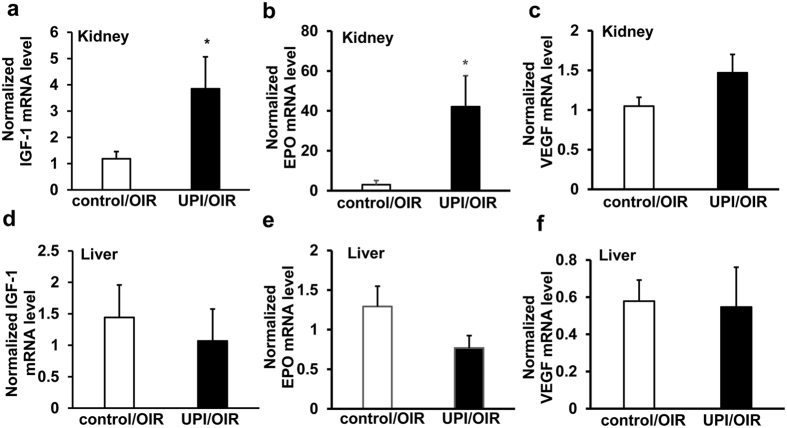
UPI pups have increased renal growth factors in OIR. Quantitative real-time PCR of (**a**) IGF-1 mRNA, (**b**) EPO mRNA and (**c**) VEGF mRNA in kidney, and (**d**) IGF-1 mRNA, (**e**) EPO mRNA and (**f**) VEGF mRNA in liver from control/OIR and UPI/OIR pups at postnatal day 18.5 (*p < 0.05, vs. control/OIR; n = 9–10).

An effect of maternal UPI is ischemia to the developing fetus, which may lead to hypoxia-induced upregulation of angiogenic factors, such as erythropoietin (EPO)^21^,^22^ and vascular endothelial growth factor (VEGF)^23^. Growth factors, including VEGF and EPO, facilitate physiologic retinal vascular development when delivered during hyperoxia, and EPO facilitates physiologic retinal vascular development during fluctuations in oxygenation, but prior to relative hypoxia, in other models of oxygen-induced retinopathy^24^,^25^. In UPI/OIR, serum EPO, but not VEGF, was significantly increased compared to control/OIR (Fig. 6b,c). We also analyzed liver and kidney growth factor expression because IGF-1 is produced in the adult rat liver^26^, and EPO is produced by kidney in the adult but by the liver in newborn infants. Renal IGF-1 (Fig. 7a) and EPO (Fig. 7b) mRNAs were increased, and a trend for increased VEGF expression (Fig. 7c) was noted in kidney but not in the liver (Fig. 7d–f). There were no differences in serum factors or expression levels of these factors in kidney or liver between male and female pups. Altogether, our data support the notion that catch-up growth, and potentially physiologic angiogenesis, may in part be due to increased hypoxia-regulated angiogenic factors.

### Association of Maternal and Pup Growth Factors

**Figure 8:**
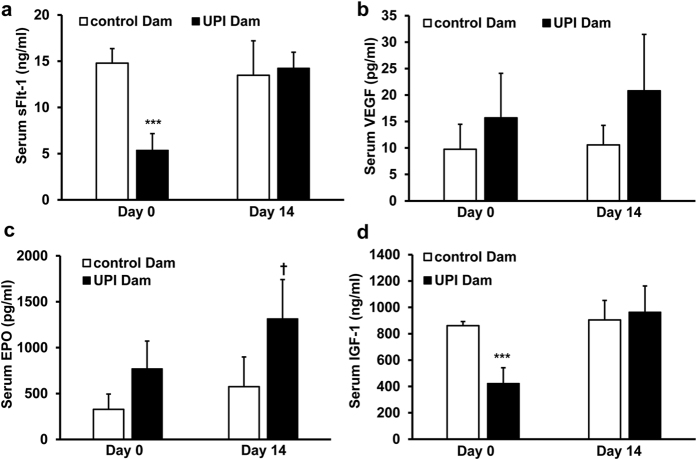
UPI dams do not show increased circulating growth factors at the time of pup birth. (**a**) ELISA of serum soluble Flt-1 (sFlt-1), (**b**) ELISA of serum VEGF, (**c**) ELISA of erythropoietin (EPO) and (**d**) ELISA of IGF-1 in control and UPI dams at the time of pup birth (Day 0) or 14 days after pup birth (Day 14) (***p < 0.001 vs. control Dam at Day 0; ^†^p < 0.05 vs. control Dam at Day 14; n = 3).

In the human condition, preeclampsia, soluble anti-angiogenic factors have been measured in the maternal circulation^27^. These findings support a theory that interference with fetal angiogenesis may contribute to infant disease and growth restriction^28^,^29^. We probed into whether UPI increased maternal soluble Flt-1 (sFlt-1) that would bind VEGF and thereby reduce circulating VEGF. Contrary to the predicted increase in sFlt-1, sFlt-1 was reduced at the time of pup birth in dams that underwent UPI surgery and recovered to normal levels 14 days after pup birth (Fig. 8a) when there was also no statistical difference in VEGF between control dams and those that underwent UPI surgery (Fig. 8b). There was no difference in serum VEGF at day 0 between pups born to dams that underwent UPI vs control (Supplemental Fig. 4). There was also no effect of UPI on room air raised pup sFlt-1 at postnatal day 14, when pup VEGF was increased (Supplemental Fig. 4). We found no significant difference between circulating EPO in dams that underwent UPI surgery and controls at the time of pup birth even though it was increased 14 days later (Fig. 8c). Dams that underwent UPI surgery had reduced IGF-1 at the time of pup birth compared to control (Fig. 8d), whereas pups had increased levels of IGF-1 (Figs 6 and 7). Our results, therefore, do not align with the notion that maternal EPO, VEGF, IGF-1 or s-FLT-1 contributed to the circulating levels found later in pups in this model.

## Discussion

The clinical literature is controversial regarding preeclampsia as either a risk or a protective factor for ROP. We sought to study the effects of maternal UPI, which is a component of preeclampsia, on full-term pups exposed to extremes of oxygen fluctuations similar to transcutaneous oxygen levels of premature infants who develop severe ROP^30^,^31^. We removed the confounding factor of premature birth, which is closely linked to both maternal preeclampsia and infant ROP^6^ and developed a composite model in which newborn, but not premature, pups born to dams with late-gestation UPI, were placed into an OIR model, which is highly representative of human ROP^11^. We tested the hypothesis that UPI increases the severity of retinal vascular features in ROP. UPI from preeclampsia in humans^3^ or from bilateral uterine artery ligation in the rat model^12^, caused extrauterine growth restriction. Extrauterine growth restriction is associated with severe ROP, and growth restriction also occurred in pups in OIR^4^.

As anticipated, UPI caused growth restriction in room air compared to control pups in room air. We anticipated that pups born to dams with UPI would have further growth restriction and, therefore, more severe features of retinopathy in OIR. Instead, pups born to dams with UPI and placed into OIR had the same weight gain as control/OIR pups, and exhibited less severe features of OIR with reduced AVA and a pattern of reduced IVNV compared to control/OIR.

Low circulating IGF-1 has been linked to increased ROP risk and reduced postnatal growth. In the UPI/OIR pups, there was increased circulating IGF-1 compared to control/OIR pups at p18.5, and this may have contributed to catch-up growth in the UPI/OIR pups. However, despite poor postnatal growth, the UPI/RA pups had increased serum IGF-1 levels compared to control/RA pups at p18.5. This finding also differed from a study in which litter sizes were smaller and only pups born less than 6 grams birth weight were analyzed^32^. In that study, IGF-1 was reduced in the growth restricted pups compared to control. Altogether, the outcomes suggest that litter size and birth weight may be contributing factors to the differential expression levels of IGF-1.

Our growth-restricted UPI/RA pups had increased physiologic retinal vascularization, a feature that aligns with the finding of increased circulating IGF-1. Previously, large litter size was reported to be associated with low circulating IGF-1 and worse retinopathy in pups in an oxygen-induced retinopathy model^33^. Another model in which protein malnutrition, rather than UPI, was used to create intrauterine growth restriction in rat pups, yielded increased, rather than reduced, avascular retina, but IGF-1 was not measured^10^. IGF-1 supplementation is being tested in clinical trials^17^ to reduce ROP. The premise of the studies is that preterm infants born before the third trimester do not have IGF-1 maternal reserve present and are not able to produce IGF-1. The fact that premature birth was specifically excluded in our study makes the IGF-1 outcomes from UPI/OIR and control/OIR models less translatable to the preterm infant with ROP, but does give insight into the effects of UPI on offspring exposed to oxygen stresses. IGF-1 is important in infant growth and different levels of IGF-1 may also help explain growth parameter variability of full-term infants born to mothers with UPI and variability in ROP of preterm infants born to mothers with UPI.

Since UPI causes placental ischemia and reduces oxygen to the fetus, we asked the question if UPI affected the expression of angiogenic factors, which are upregulated in hypoxia and important for retinal vascularization and potentially neuronal protection. UPI/OIR pups had increased circulating and renal EPO, but not VEGF, compared to control/OIR, suggesting that perhaps maternal placental ischemia led to increases in newborn renal and circulating angiogenic factor, EPO, in association with greater physiologic retinal vascularization and reduced pathologic vasoproliferation, an observation that we previously made^34^. However, our results did not support the hypothesis that maternal EPO, VEGF, IGF-1 or sFlt-1 contributed to the circulating levels found later in pups or of VEGF at the time of pup birth. It remains unknown if other circulating maternal factors transfer to pups at birth and affect outcomes. The ligation may impair some transfer of circulating factors between the mother and fetus in this particular model, and more study would be needed to address the hypothesis that maternal factors transferred to the fetus affect fetal development and pathology.

The different effects of hypoxia on VEGF and EPO may be due to differential activation of hypoxia-induced factor-1 (HIF-1) or 2 (HIF-2) in UPI/OIR pups. VEGF is regulated by HIF-1^35^, whereas EPO is a target gene of HIF-2^36^. There is evidence to support the importance of EPO in our findings. A form of tissue protection by EPO has been described in the cardiovascular and cerebrovascular literature^37^. Repetitive brief periods of ischemia have been shown to have protective effects on the myocardium by reducing infarct size^38^ and preserving vascular function^39^,^40^. In addition, EPO has been shown to be neuroprotective, and its induction is believed to have a protective role in cerebral ischemia and after hypoxic reconditioning^41^. Since UPI causes placental ischemia and reduces oxygen, it is plausible that intermittent hypoxia/ischemia to the fetus may be protective through a preconditioning mechanism. Human infants with intrauterine growth restriction have increased circulating EPO and even polycythemia^42^. EPO can support vascularization in developing organs, including the retina^21^,^34^, and at least in some preterm infants may, therefore, provide protection against ROP. This finding may help explain some of the variability in ROP severity seen in premature infants. However, EPO has been associated with worse retinopathy both experimentally^43^,^44^ and clinically^45^ depending on when it is administered. A clinical trial currently testing the role of EPO in premature infant cognitive development may provide clarity as to its effects on ROP^46^.

We modified the OIR model to include two 24-hour cycles of 50% oxygen in order to reduce pup mortality. We strove to keep other parameters the same between control/OIR and UPI/OIR pups by optimizing the model to have litters with comparable final litter sizes while accounting for effects from clustering of pups by statistical analysis using multivariable mixed effects linear regressions. However, we cannot rule out a bias of selection of the hardiest pups with improved survival in the UPI group. In nature, infants born to mothers with preeclampsia and UPI do not always survive. Very premature and small for gestational age infants have higher mortality, lower insulin and growth factor secretion, and diminished antioxidant capacity^47^ than counterparts with appropriate for gestational age birth weights. Reduced antioxidant capacity and growth factors also predispose the premature infant to severe ROP^48^,^49^. Our model may select out pups that can produce factors, such as EPO and IGF-1, to enhance retinal vascularization and growth in the setting of OIR and UPI. The study reflects the complexities when studying preeclampsia, which can lead to a range of levels of UPI severity that affect the fetus.

In conclusion, we found that pups born to dams with severe UPI, in the absence of preterm birth, and exposed to fluctuations in oxygenation that cause retinal vascular features similar to human ROP, appear to have a growth advantage in association with increased growth factors, increased physiologic retinal vascularization, and a trend toward reduced IVNV. Our findings support a hypothesis that severe UPI in mothers may upregulate hypoxia-induced angiogenic factors in some infants who are able to produce these factors to increase physiologic retinal vascularization and reduce severity of ROP, potentially also increasing infant survival. However, premature infants often have lower reserve and may not be able to produce sufficient factors, including IGF-1. More study of the levels of circulating angiogenic factors in preterm infants may be considered to refine dosing regimens for EPO or anti-VEGF agents.

## Methods

### Animals and Ethical Statement

All methods were carried out in accordance with the Guidelines and Regulations for the Care and Use of Laboratory Animals by the University of Utah (Salt Lake City, Utah) and the Association for Research in Vision and Ophthalmology Statement for the Use of Animals in Ophthalmic and Vision Research. All animal experimental protocols were approved by Institutional Animal Care and Use Committee of University of Utah. Timed-pregnant Sprague-Dawley rats were purchased from Charles River (San Diego, California). Animals had access to food and water ad libitum and were kept on a 12/12 light-dark cycle.

### Bilateral uterine artery ligation

Bilateral uterine artery ligation was used as a model for uteroplacental insufficiency (UPI) to cause pup intrauterine growth restriction, as previously described^12^. On embryonic day 19.5, pregnant dams were anesthetized using ketamine (60 mg/kg), xylazine (12 mg/kg) and buprenorphine (0.025 mg/kg). The abdominal wall was opened; both uterine horns were placed outside the body cavity, and the uterine arteries were exposed. Both uterine arteries were partially tied off with silk sutures to reduce, but not completely block, blood flow to the fetuses. (All surgeries were performed by the same two experienced surgeons). The uterine horns were placed back inside the dam and the abdominal wall was sutured. Dams were given access to carprofen wafers for post-surgical pain relief. In control dams, anesthesia alone was carried out, which was reported to cause similar growth parameters on pups as sham surgery^19^. Dams were allowed to deliver pups naturally, usually 2–3 days later after a gestational period of 21–23 days. Pups were weighed immediately after birth and individually marked by paw tattoos. Litter sizes were maintained at 12–16 pups by supplementing pups from other litters. Therefore, some OIR litters had pups from UPI and control dams. However, for analysis pups were divided into control or UPI pups.

### OIR model

In order to reduce pup mortality, a modification of the previously described 50/10 OIR model was used^9^,^50^. Dams with their litters were transferred into an OxyCycler (Biospherix, New York) within 6 hours of birth. Oxygen concentrations were maintained at 50% O_2_ for the first 48 hours instead of for the first 24 hours as in the standard 50/10 OIR model, and subsequently cycled between 10% and 50% O_2_ until postnatal day (p)15. Litters were removed from the OxyCycler and raised in room air for an additional 3.5 days. Pups were euthanized on postnatal day 18.5 by intraperitoneal injection of ketamine (70 mg/kg) and xylazine (8 mg/kg). (This also differed from the standard 50/10 OIR model, in which analysis is performed at day 18 or day 20).

### Retinal flat mount preparation, imaging and analysis of avascular retinal area (AVA) and intravitreal neovascularization (IVNV)

After enucleation, the cornea was pierced with a 30-gauge needle, eyes were fixed in 4% paraformaldehyde (PFA) for 1 hour and placed into PBS (containing in mM NaCl 137, KCl 2.7, Na_2_HPO_4_ 8, KH_2_PO_4_ 2, pH 7.4). The retina was dissected after removing the cornea, lens and vitreous followed by separation of RPE, choroid and sclera and flattened onto slides. The retinal flat mounts were stained with Alex-Fluor 568-labelled isolectin GS-IB4 from Griffonia simplicifolia (2.5 μg/mL, Invitrogen, Grand Island, New York) to stain the retinal vasculature. Flat mounts were imaged at 10x magnification using an inverted fluorescence microscope (Olympus IX81, Oympus Corp. Tokyo, Japan), and individual images were joined using Metamorph 7.0 software (Molecular Devices Inc., Sunnyvale, California). The percentages of avascular retina area/total retinal area and of intravitreal neovascular area/total retinal area were determined by two masked reviewers using Image J software (National Institutes of Health, Bethesday, Maryland) and defined as AVA and IVNV, respectively.

### Determination of serum angiogenic factor concentrations by ELISA

Whole blood was collected, allowed to clot on ice and spun down at 2000 × g for 30 min at 4 °C. Serum samples were collected and stored at −80 °C until use. Serum concentrations of VEGF, EPO, IGF-1 and soluble Flt-1 were determined by ELISAs using Rat VEGF, mouse erythropoietin and Rat IGF-1 Quantikine ELISA kits (R&D Systems, Minneapolis, Minnesota), and rat soluble Flt-1 ELISA kit (LifeSpan BioSciences, Seattle, WA) according to the manufacturers’ instructions.

### RT-PCR of angiogenic and growth factors in liver and kidney

Kidney and liver tissue samples were homogenized by sonication in 150 μl RLT buffer and mRNA was isolated using RNeasy Mini Kit (Qiagen, Valencia, CA, USA). 500 ng mRNA was reverse transcribed in a total volume of 20 μl using High Capacity cDNA Reverse Transcription Kit (Applied Biosystems, ThermoFisher Scientific). Two-microliter cDNA were used in a total volume of 20 μl to determine gene expression using Taqman gene expression assays for rat VEGF, EPO and IGF-1, as well as the housekeeping genes GAPDH and TBP. mRNA expression of the target genes was normalized to that of the housekeeping genes and is displayed as 2^−ΔΔCT^.

### Statistical analysis

In the present study we used pups from five to six litters per experimental group, leading to potential clustering effects. To test the hypothesis that UPI has a detrimental effect on vascular features of OIR, we compared UPI pups to control pups using multivariable mixed effects linear regressions, thus adjusting for the potential confounder of pups being born and raised in the same litters. In order to determine whether vascular outcomes of OIR were dependent on birth weight or weight gain, Pearson correlations were computed and slopes compared between control and UPI pups. Statistical analysis was performed using ANOVA with post hoc protected corrections using the Bonferroni Multiple Comparison Test. Multivariable mixed effects linear regressions and Pearson correlations were performed using STATA14 software (Strata Corp. College Station, Texas). Results were mean ± SEM. A minimum *P* value of <0.05 was considered statistically significant.

## Additional Information

**How to cite this article**: Becker, S. *et al*. Protective effect of maternal uteroplacental insufficiency on oxygen-induced retinopathy in offspring: removing bias of premature birth. *Sci. Rep.* **7**, 42301; doi: 10.1038/srep42301 (2017).

**Publisher's note:** Springer Nature remains neutral with regard to jurisdictional claims in published maps and institutional affiliations.

## Supplementary Material

Supplemental Figures
