# Supplementary material for: Protective effect of maternal uteroplacental insufficiency on oxygen-induced retinopathy in offspring: removing bias of premature birth
Source: Sci Rep. 2017 Feb 14;7:42301. doi: 10.1038/srep42301 (PMC5307308; doi:10.1038/srep42301)
Supplement: Supplemental Figures [file srep42301-s1.pdf]

Title: Protective effect of maternal uteroplacental insufficiency on oxygen-induced retinopathy in offspring: removing bias of premature birth

Authors: Silke Becker[1], Haibo Wang[1], Baifeng Yu[2], Randy Brown[1], Xiaokun Han[1], Robert H. Lane[3], M. Elizabeth Hartnett[1]

Affiliations: [1]John A. Moran Eye Center, University of Utah, Salt Lake City, Utah, USA; [3] Children's Hospital of Wisconsin, Milwaukee, Wisconsin, USA.

Corresponding author:

M. Elizabeth Hartnett, MD.

Address: 65 N. Mario Capecchi Drive, Salt Lake City, UT 84132.

Tel: 801-213-4044; Fax: 801-581-3357

Email: ME.Hartnett@hsc.utah.edu

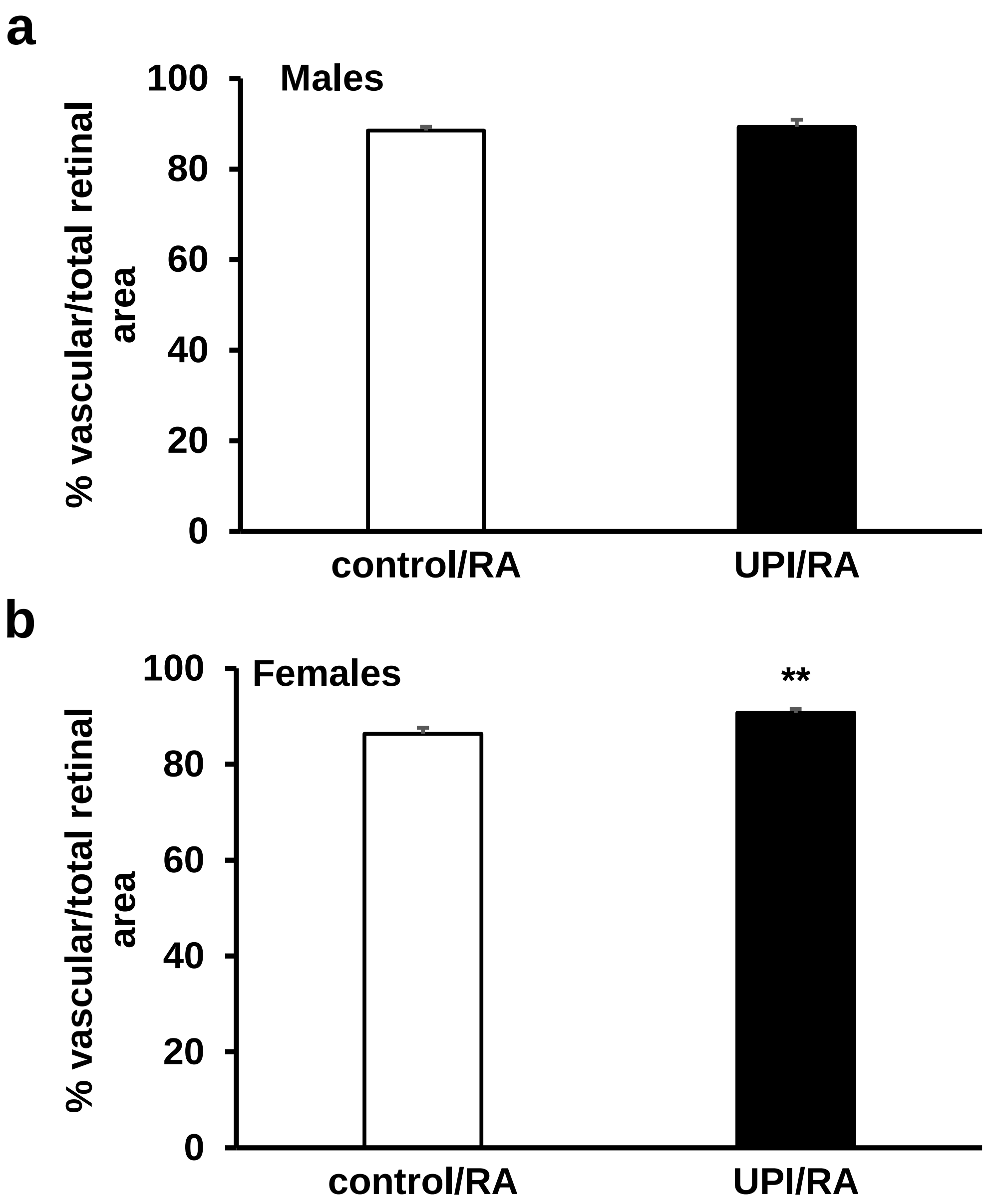

Supplemental Figure 1: Vascularized retinal area/total retinal area (%) in (a) Male and (b) Female Control/RA and UPI/RA pups at postnatal day 7.

Supplemental Table 1: Definitions

control/RA	Pups born to dams exposed to anesthesia and raised in room air
UPI/RA	Pups born to dams with UPI after bilateral uterine artery ligation and raised in room air
control/OIR	Pups born to dams exposed to anesthesia and OIR (Figure 3)
UPI/OIR	Pups born to dams with UPI after bilateral uterine artery ligation and OIR (Figure 3)

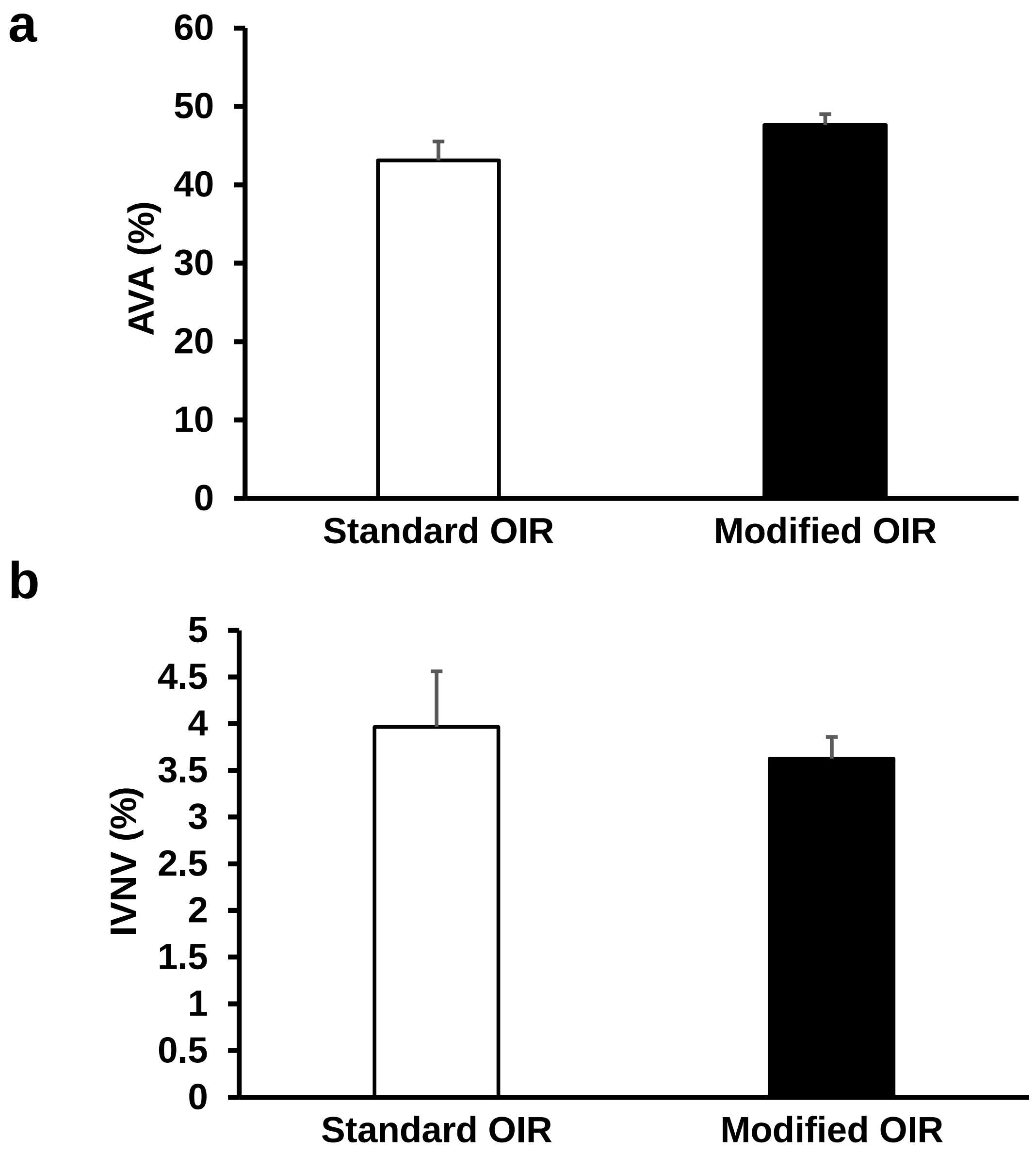

Supplemental Figure 2: Quantification of (a) AVA and (b) IVNV in models of standard OIR and modified OIR pups at postnatal day 18.5

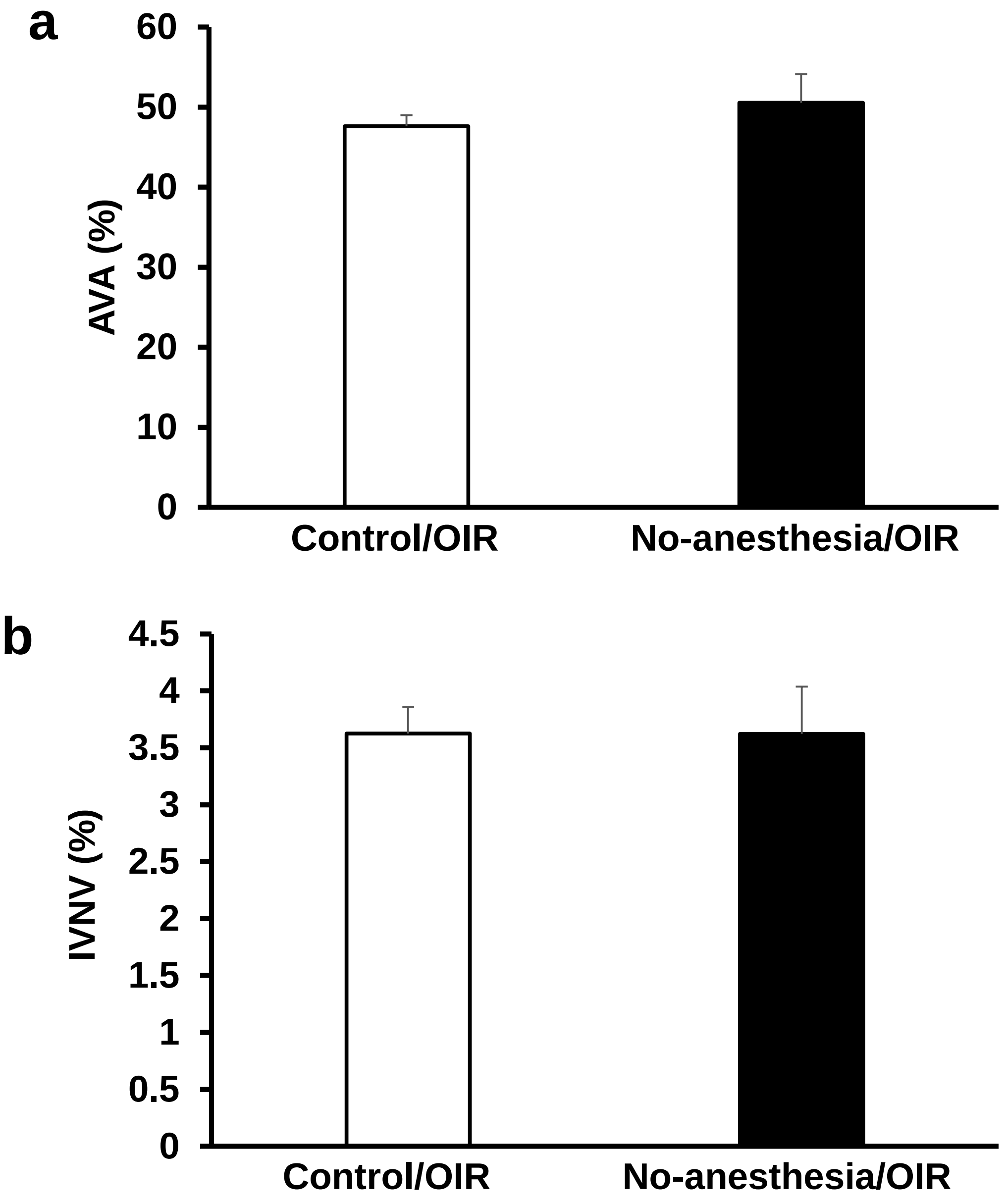

Supplemental Figure 3: Quantification of (a) AVA and (b) IVNV in Control/OIR and No-anesthesia/OIR pups at postnatal day 18.5

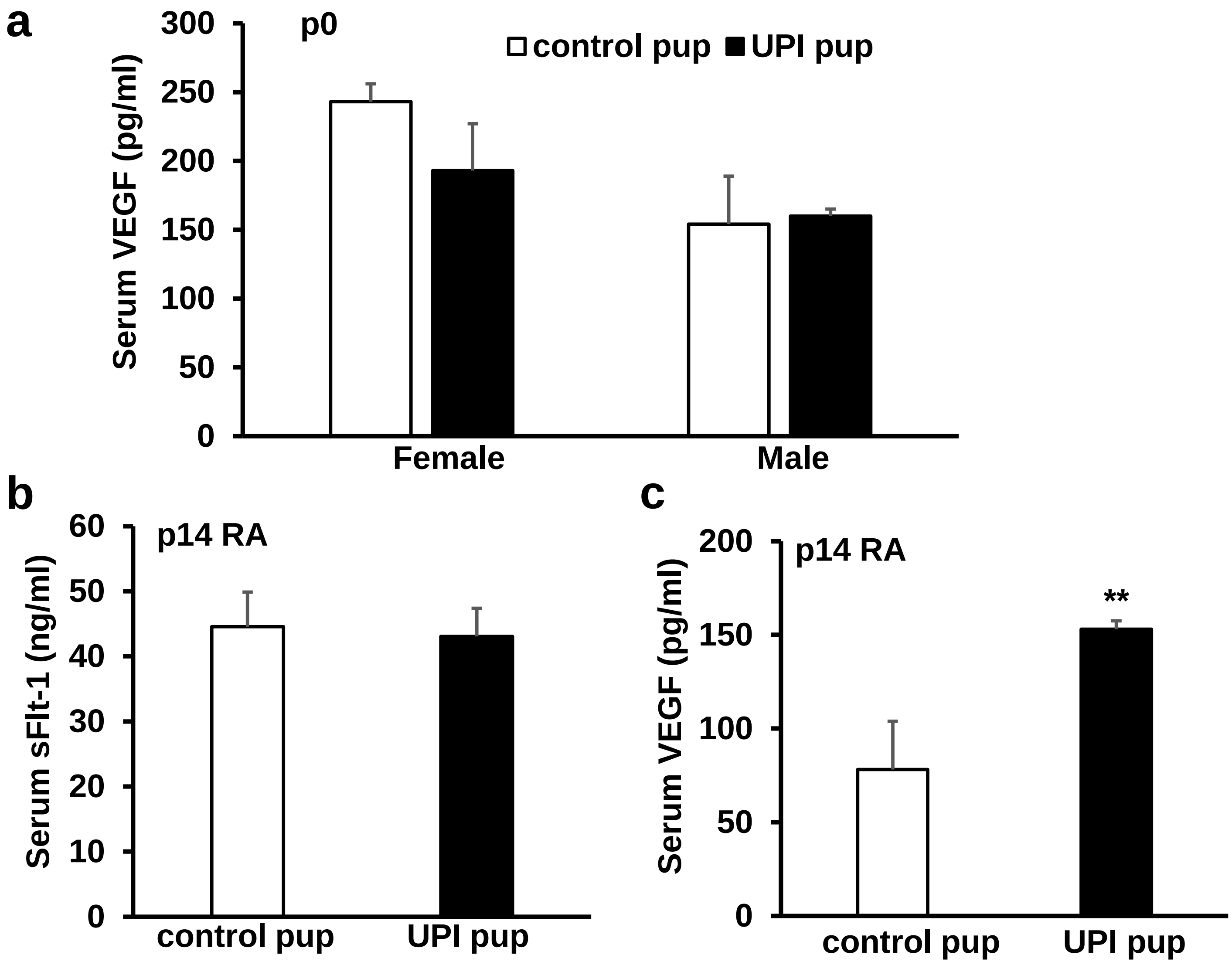

Supplemental Figure 4. (a) ELISA of serum VEGF in pups born to control or UPI dams at birth (p0), (b) ELISA of serum soluble Flt-1 (sFlt-1) and (c) ELISA of serum VEGF in room air pups at postnatal day 14 (p14RA) born to control or UPI dams (**p<0.01 vs. control pup).
